# Zinner's Syndrome: A Rare Diagnosis of Dysuria Based on Imaging

**DOI:** 10.1155/2020/8826664

**Published:** 2020-12-09

**Authors:** Ahmed Ibrahimi, Abdelmoughit Hosni, Idriss Ziani, Fatima Zahra Laamrani, Hachem El Sayegh, Laila Jroundi, Lounis Benslimane, Yassine Nouini

**Affiliations:** ^1^Department of Urology A, Ibn Sina University Hospital, Rabat, Morocco; ^2^Faculty of Medicine and Pharmacy, Mohammed V University in Rabat, Morocco; ^3^Emergency Radiology Department, Ibn Sina University Hospital, Rabat, Morocco

## Abstract

Zinner's syndrome is a rare congenital malformation of the seminal vesicle and ipsilateral upper urinary tract, due to developmental arrest in early embryogenesis of the Müllerian duct. Clinical presentation is nonspecific and includes voiding symptoms such as dysuria, ejaculatory disorders, and hypogastric or perineal pain. The diagnosis is made with imaging techniques, notably Magnetic Resonance Imaging (MRI) which remains the gold standard exam for diagnosis confirmation and therapeutic management. Treatment options depend on the severity of symptoms, the size of the cyst, and the complications. Herein, we report a rare case of a 33-year-old young patient who presented recurrent dysuria and ejaculatory disorders for the last 5 years. Imaging studies revealed an empty left renal fossa, with cystic pelvic mass related to the seminal vesicle and which was compatible with the diagnosis of Zinner's syndrome. The patient underwent successful laparoscopic removal of the cyst and seminal vesicle, with total disappearance of urinary and sexual complaints with a 3-year follow-up.

## 1. Introduction

Zinner's syndrome is a rare congenital malformation of the seminal vesicle and ipsilateral upper urinary tract [1, 2]; it is characterized by the simultaneous association of unilateral renal agenesis, ipsilateral seminal vesicle cyst, and ipsilateral ejaculatory duct obstruction. It is often diagnosed in the second and third decades of life and can lead to serious complications, particularly infertility [3, 4]. Clinical presentation is nonspecific, thus often making the diagnosis delayed or missed, and the majority of patients remain asymptomatic until beginning sexual activity [1]. Magnetic Resonance Imaging (MRI) occupies a prominent place in the diagnostic arsenal and remains the examination of choice to make the diagnosis [5].

We herein, through this rare case of a 33-year-old man diagnosed with Zinner's syndrome, aimed to discuss the hierarchy and the results of different imaging techniques that are employed in order to reach the correct diagnosis, as well as the different clinical, etiological, and therapeutic aspects of this uncommon condition.

## 2. Case Presentation

A 33-year-old patient, father of three children, without past medical or surgical history presented to the urology department for apyretic recurrent dysuria, with episodes of hematospermia and painful ejaculation evolving for 5 years.

Physical examination revealed no palpable pelvic mass, with normally developed external genitalia. Digital rectal examination (DRE) was painless and found a renitent mass facing the anterior wall of the rectum, with normal prostate volume and consistency. Routine blood and urine laboratory tests, including urine culture, were normal.

**Figure 1:**
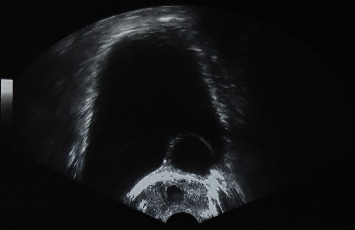
Transrectal ultrasonography showing a large left retrovesical cyst compressing the urinary bladder and protruding into the bladder lumen.

Abdominal ultrasonography revealed an empty left renal fossa, associated with a left retrovesical liquid mass. A transrectal ultrasonography was performed, showing a large left retrovesical hypoechoic mass with a posterior enhancement and impure content, compressing the urinary bladder and protruding into the bladder lumen (Figure 1).

**Figure 2:**
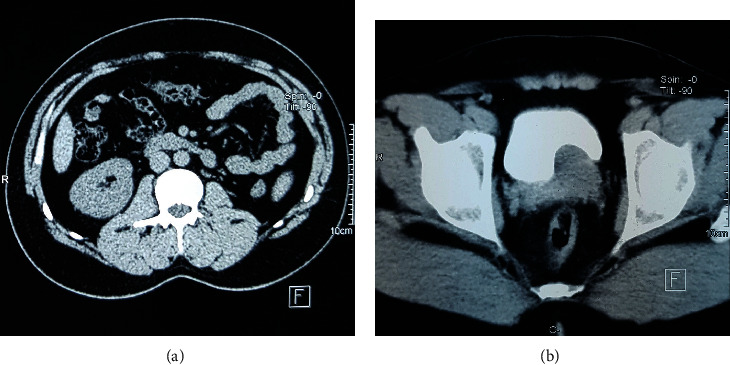
(a) Axial view of CT scan showing a single right kidney with empty left renal fossa; (b) CT scan of the pelvis revealing a homogeneous liquid mass retrovesical compressing the urinary bladder and protruding into the bladder lumen.

Abdominal and pelvic computed tomography (CT) showed left renal agenesis (Figure 2(a)), associated with the presence of a large left retrovesical mass without contrast enhancement, suggesting a cystic mass and compressing the posterior bladder wall (Figure 2(b)).

**Figure 3:**
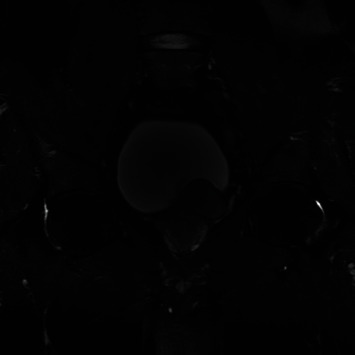
MRI coronal image in the T2-weighted sequence showing a seminal vesicle lesion which is separated from the prostate by its capsule.

**Figure 4:**
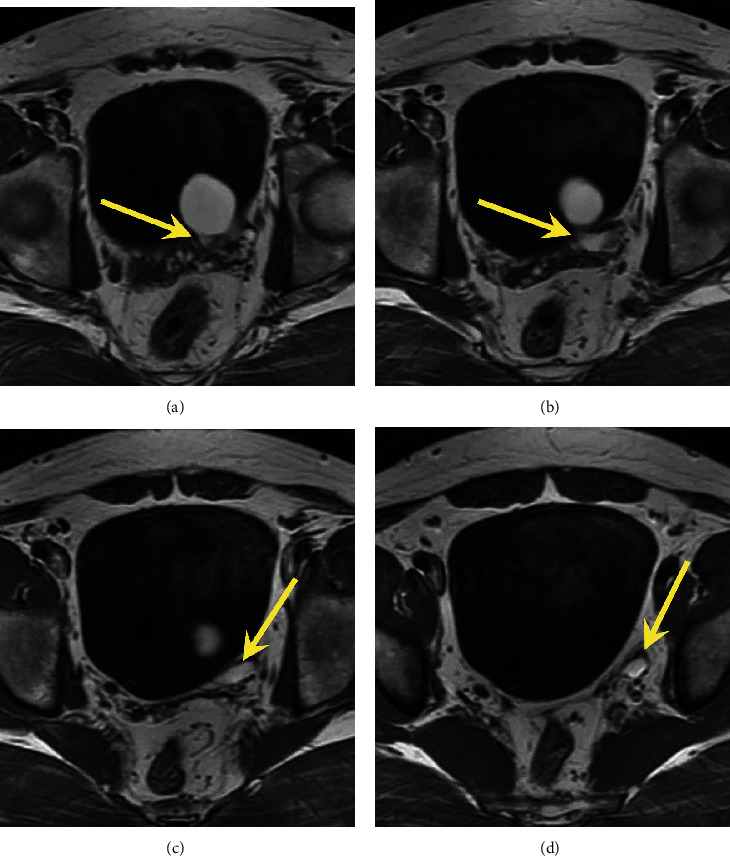
MRI axial superposed images on the seminal vesicle cyst in the T1-weighted sequence. Yellow arrow showing the tubular structure linked to the seminal vesicle cyst considered an ectopic ureter.

**Figure 5:**
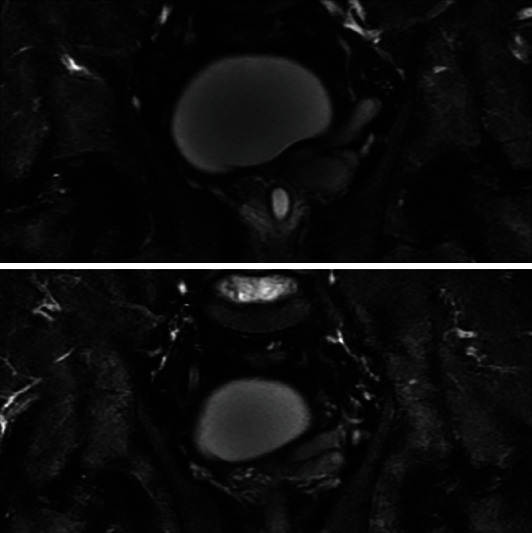
MRI coronal slices showing the communication between the seminal vesicle cyst and ectopic ureter.

A pelvic MRI was performed to identify precisely the origin of the cystic mass using a T1-weighted, T1-weighted with gadolinium injection, and T2-weighted sequences in the axial and coronal plans (Figures 3 and 4). We found then a unique cystic-to-tubular structure in the seminal vesicle appearing hyperintense on T2W images and hyperintense on T1W images (due to hemorrhage and a high proteinaceous concentration on the seminal fluid), measured to 20 × 35 × 40 mm. From its posterolateral side emerges a tubular structure crossing the iliac vessels anteriorly, which was considered an ectopic ureter (Figures 4 and 5). The right seminal vesicle did not show any abnormality. CT findings were reviewed, and the presence of the ectopic ureter was noticeable within the multiplanar reconstructions.

**Figure 6:**
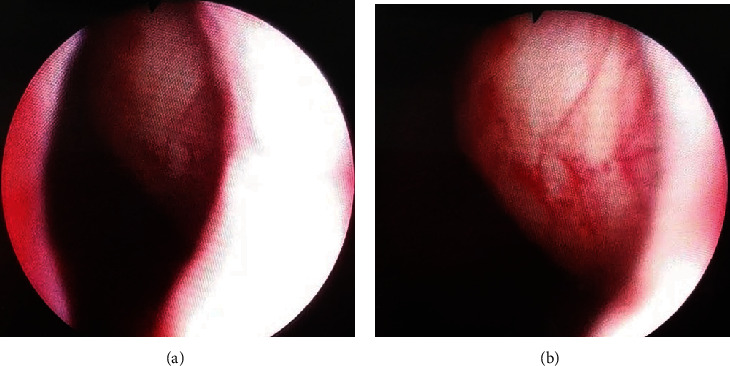
(a, b) Urethrocystoscopy showing a large intraluminal protruding mass at the left lateral trigonal area and partially obstructing the bladder neck.

Urethrocystoscopy showed no lesions in the urethra or bladder, revealing a large intraluminal protruding mass at the left lateral trigonal area and partially obstructing the bladder neck (Figure 6).

Imaging findings and cystoscopic examination lead to the diagnosis of Zinner's syndrome, and surgical management was planned.

The patient underwent laparoscopic removal of the cyst and seminal vesicle. The postoperative course was uneventful, and the patient was discharged from the hospital on the 4th day without any complication. The follow-up was marked by total disappearance of urinary and sexual complaints.

## 3. Discussion

Zinner's syndrome was first described in 1914 by Zinner. It is defined as the association of a seminal vesicle cyst, an ipsilateral renal agenesis, and an ejaculatory duct obstruction [1, 4]. The ipsilateral ureter can be absent or incomplete or may have an abnormal course towards the seminal vesicle. This last situation, which was found in our case, concerns 36% of the cases [5, 6]. Zinner's syndrome remains a rare congenital malformation with only 200 cases reported in the available literature [1, 4, 5]. The association of abnormalities is frequently found on the right side, with a right-sided ratio of 2 : 1. Bilaterality is found in only 2% of cases [7]. Otherwise, a contralateral renal agenesis was reported in 4 cases of seminal vesicle cysts [8].

Symptoms depend mainly on the cyst's size; commonly, cysts less than 5 cm are asymptomatic. Symptoms are usually present at the second or third decade, with a mean age at presentation of 30 years; this age corresponds to a significant increase in the size of the cyst, as well as the beginning of sexually active life. Symptoms reported in the literature are usually related either to micturition which includes dysuria, obstructive urination, frequency, urgency, and hematuria; to perineal, scrotal, hypogastric, or defecation pain; or in some cases to ejaculatory disorders such as pain following ejaculation, infertility, and hematospermia. Complications such as epididymitis or prostatitis represent a considerable form of revelation [6, 7, 9]. Rarely, giant cysts can lead to bladder or colon obstruction [5].

Infertility is frequently associated; its pathogenesis is unclear, but it is more likely due to the associated ejaculatory duct obstruction. Examination of seminal fluid should be systematic in the presence of Zinner's syndrome; results can show a low ejaculatory volume, azoospermia, alkaline pH, low concentration of carnitine and fructose in the seminal plasma, and high citrate level [7, 9]. In our patient, the symptoms were troublesome despite the small size of the cyst, which did not exceed 4 cm, probably because the cyst is protruding in the bladder lumen and thus causing voiding complaints; fertility was conserved in our patient, and none of the cited complications did happen.

Imaging remains the key to diagnosis. Transrectal ultrasonography (US) shows the seminal vesicle cyst as an anechoic pelvic mass with a thick wall; infection or hemorrhage from the cyst may appear as cystic mass containing internal echos [5].

CT is superior to ultrasonography, and reported findings are of a retrovesicular cystic pelvic mass arising from an enlarged seminal vesicle associated with ipsilateral renal agenesis, but it may be insufficient to confirm the diagnosis [1, 3].

MRI is considered superior to CT scan in the analysis of these conditions [5]. With its invasiveness, multiple pulse sequences, multiplanar capability, and high soft-tissue resolution, it is considered a “gold standard” for diagnosis and surgical planning. Images from MRI provide a better characterization of the cyst content, a precise detection of associated genitourinary abnormalities, and an evaluation of the anatomic relationship with the pelvic structures. Unilocular cystic masses are more commonly found than tubular and multilocular features. Typically, the cyst content is described as a homogeneous hyposignal on T1-weighted sequences and homogeneous hypersignal on T2-weighted sequences. However, some cysts may have a hypersignal on both T1- and T2-weighted sequences related to an old hemorrhage and a high proteinaceous concentration on the seminal fluid [1, 10]. Intracystic vegetations can be a sign of malignancy, although malignant degeneration remains exceptional with only 3 reported cases in the literature [11, 12].

The differential diagnosis of pelvic cysts in male patients includes prostatic utricle cystic dilatation, prostatic utricle cysts (previously known as Müllerian duct cysts), ejaculatory duct cysts, prostatic cysts, diverticulosis of the ampulla of the deferens canal, ectopic ureterocele, and abscess [13]. Diagnosis criteria of seminal vesicle cysts are the cyst position (median, paramedian or lateral, and intra- or extraprostatic), associated abnormalities (renal agenesis, anomalies of the external genitalia), and imaging aspects on MRI [14].

Once diagnosed, management of seminal vesicle cysts depends on their size and symptomatology, as well as the presence of complications [2, 4]. A wide variety of treatment modalities and techniques have been reported including conservative management represented by percutaneous drainage and transurethral or transrectal aspiration, but both are associated with a greater risk of recurrence [3]. Surgical procedures are the mainstay in the management of symptomatic, complicated, or recurrent patients based on open surgery or minimally invasive surgery, with laparoscopic transperitoneal approaches or, recently, robotic-assisted approaches which guarantee a successful outcome [2, 4, 6]. Nevertheless, the challenge of different treatment approaches is preserving the fertility of the patient and relieving the symptoms.

In our case, laparoscopic removal of the cyst and seminal vesicle was performed after failure of transrectal ultrasonography-guided aspiration of the cyst, which was complicated by hemorrhage, with inability to evacuate the cyst content completely. Conservative treatment was attempted in our patient 6 months ago, and since then, the cyst has increased in size and the symptoms became worse, affecting the patient's quality of life, thus requiring recourse to surgical treatment.

## 4. Conclusion

Zinner's syndrome is an uncommon cause of dysuria in men; its diagnosis is based on imaging techniques. Ultrasonography is a noninvasive exam and could provide valuable information, CT scan could be sufficient to make the diagnosis if it defined the origin of the pelvic mass, and MRI is the exam of choice for a precise lesional statement and successful therapeutic management.

## Data Availability

The datasets used and/or analysed during the current study are available from the corresponding author on reasonable request.
